# Effects of Aquatic and Dry Land Resistance Training Devices on Body Composition and Physical Capacity in Postmenopausal Women

**DOI:** 10.2478/v10078-012-0035-3

**Published:** 2012-05-30

**Authors:** Juan Carlos Colado, Xavier Garcia-Masso, Michael E Rogers, Victor Tella, Juan Benavent, Estelio H Dantas

**Affiliations:** 1 University of Valencia, Laboratory of Physical Activity and Health, Department of Physical Education and Sports, Valencia, Spain.; 2 Department of Human Performance Studies, Center for Physical Activity and Aging, Wichita State University, Wichita, Kansas..; 3 Biosciences Doctoral Program, Federal University of State of Rio de Janeiro, Rio de Janeiro, Brazil.; 4 Invited Researcher, University of Valencia, Valencia, Spain.

**Keywords:** aquatic training, weight machines, elastic bands, fitness

## Abstract

To determine the effects of a supervised strength training program on body composition and physical capacity of older women using three different devices: weight machines, elastic bands, and aquatic devices that increase drag forces (ADIDF). Four groups were formed: control group, weight machine group (WMG), elastic band group (EBG) and a group that used ADIDF (ADIDFG). Body composition and physical capacity were assessed before and after the intervention period. The ADIDFG showed improvements in fat mass (FM), fat-free mass of the left arm (FFM-LA) and right arm (FFM-RA), knee push-up test (KPT), squat test (ST) and crunch test (CT) (p <0.05). Individuals in the EBG and WMG also improved their FM, fat free mass (FFM), FFM-LA, FFM-RA, KPU, ST and CT. ADIDF training improves body composition and physical capacity of postmenopausal women as does performing land-based training programs.

## Introduction

The aging process is usually associated with changes that result in increased body fat and a loss of fat-free mass (FFM) (Colado et al., 2009). These changes are some of the major public health problems affecting women and their critical period for incidence and development is during post menopause (Bemben et al., 2000; Colado and Triplett, 2008). In addition, there is a significant relationship between reduced muscle strength, physical capacity and quality of life with the loss of FFM (Bemben et al., 2000; Katula et al., 2006). Because one of the main objectives of public health policies is to increase the life expectancy of individuals while maintaining a sufficient level of functional health (Chulvi-Medrano et al., 2009; Katula et al., 2006), it is necessary to develop various strategies to prevent the deterioration of health caused by changes that occur during the aging process.

The practice of physical activity has been one of the most widely-studied strategies to improve the quality of life on adults and older people (Colado and Triplett, 2008; Katula et al., 2006). Some researchers have found positive results when using strength training to induce increases in FFM and physical ability in older people (Bemben and Bemben, 2000; Elliot et al., 2002; Fahlman et al., 2002). The American College of Sports Medicine recommends that people who begin a program of strength training should use free-weights or weight machines (WMs) (American College of Sports Medicine, 2009). However, in many cases, it is not possible to access these devices due to a lack of facilities or financial resources. In addition, because the average dropout rate in exercise programs using these devices is approximately 50% during the first year of practice (Dishman et al., 2004), it seems important to propose alternatives to the use of WMs for strength training that might be more convenient for certain population groups, such as older women. In this context, Colado and Triplett (2008) have proposed the use of elastic bands (EBs) as an economical alternative to the use of WMs, as no significant differences between the two devices were found in terms of their effects on body composition and physical capacity in the short term.

The practice of physical activity in an aquatic environment has expanded greatly in recent decades because it is the only therapeutic and rehabilitation practice to be used in both recreational practice in healthy individuals (Colado and Triplett, 2009; Takeshima et al., 2002) and in high-performance practices (Bushman et al., 1997; Frangolias et al., 1996; Martel et al., 2005; Miller et al., 2002; Robinson et al., 2004; Triplett et al., 2009). These new fields of application are supported by short-term improvements in muscle strength, power and fat-free mass in young, physically active men after aquatic strength training using aquatic devices that increase drag force (ADIDF) (Colado et al., 2009a). Additionally, recent studies comparing the physiological improvements and the increased physical capacity resulting from training programs based on the use of ADIDF or EBs in postmenopausal women found no significant differences between the use of these various force training devices (Colado et al., 2009b).

In light of this, aquatic exercise programs employing ADIDF with the aim of improving the physical capacity and body composition of individuals could be an alternative to land-based training with EBs and WMs. However, there are no studies comparing the effects of strength training using WMs, EBs and ADIDF on these parameters in respect to which one best prevents degenerative physical changes associated with aging.

Consequently, this study aimed to determine the short-term effects of a supervised strength training program using three different devices (WMs, EBs and ADIDF) on body composition and physical capacity in postmenopausal women. Once the effects of these programs have been established, it will be possible to determine which of them is most effective, and could thus result in maximal benefits for body composition and physical fitness of postmenopausal women.

## Material and Methods

### Study design

We used a randomized, controlled and multi-group experimental design to assess the effects of resistance exercises in postmenopausal women. Four experimental groups were formed: one was the control group (CG), the second underwent training using WMs (WMG), the third used EBs (EBG) and the final group used ADIDF (ADIDFG). Body composition and physical capacity were assessed before and after the intervention period. To ensure that the three experimental groups were similar, we selected exercises with similar stabilization needs that also engaged the same agonist muscle groups. To control and equalize the intensity between the groups, we used a method based on the number of repetitions required and the OMNI perceived exertion scale for resistance exercise for active muscles (OMNI-RES-AM) (Robertson et al., 2003; Colado et al., 2011). Previous studies have described this method to control the intensity during exercises using WMs, EBs and ADIDF (Colado and Triplett, 2008; Colado and Triplett, 2009; Colado et al., 2009a; Colado et al., 2009b; García-Massó and Colado, 2010).

### Subjects

Before the beginning of the study, 92 volunteer women were examined to confirm that they were not taking medicines, were not under any hormonal therapy and were functionally independent, free from neurological, cardiovascular, metabolic, inflammatory or musculoskeletal problems that could exclude them from the study. Furthermore, it was confirmed that none of them had previously participated in a program of strength training or had completed any type of aerobic exercise in the previous four years. After the initial screening, 15 women were excluded because they did not meet the study inclusion criteria. The remaining 77 women were randomly assigned to one of the four groups, but in anticipation of potential dropouts that often occur during the administration of unpaid research studies, twice as many women were placed into each exercise group. Therefore, there were 22 women in each exercise group and 11 in the control group, with this sample size the power of the study was of 75%. Seventy-seven women began the study and 15 dropped out throughout the program due to family commitments.

Table 1 shows the composition and final characteristics of the groups. All women were housewives, had similar socioeconomic statuses and conducted similar activities in their daily lives. In addition, all women were menopausal, having been amenorrheic for at least one year before the start of the study [mean time of amenorrhea: 4.37 (2.86) years].

All subjects were informed of the training and testing, signed a written informed consent to participate, and were instructed not to modify their behavior or diet, nor to perform any other type of physical exercise for the duration of the study. To ensure strict compliance with the participation instructions, these aspects were monitored weekly by the researchers using a diary of activities and diet that was completed daily by the women. The study was approved by the institutions′ review board.

### Procedures

Measurements were made in the same week in a controlled environment at a room temperature of 22 ± 0.1°C. The body composition tests were always performed 24 hours before the muscle function tests, which were performed 72 hours after ceasing heavy exertion. For both pre- and post-tests, the subjects attended a familiarization session to learn or review, as appropriate, the techniques for performing the tests 48 hours before carrying out the first muscle function tests (Ploutz-Snyder and Giamis, 2001).

Therefore, the evaluation week consisted of the following: a test familiarization or review session (Monday), the first body composition measurements (Tuesday), the first physical capacity measurements (Wednesday), a rest day (Thursday), the second body composition measurements (Friday) and the second physical capacity measurements (Saturday). The best value for each test was used in the statistical analysis. All tests resulted in very high intra class correlation coefficients (0.90−1.0) for test-retest reliability. All measurements for testing (pre- and post-training) were made using identical equipment, positioning, test technicians and techniques for each subject. The examiners were appropriately trained and qualified.

### Body composition

A bioelectrical impedance analysis system (BC-418, Tanita Corp., Tokyo, Japan) was used to determine body composition. All subjects were evaluated following the guidelines proposed by Dixon et al. (Dixon et al., 2005) and the manufacturers. Values of body weight, body fat (FM), fat-free mass (FFM), fat-free mass in the left (FFM-LA) and right arm (FFM-RA) and fat-free mass in the left (FFM-LL) and right leg (FFM-RL) were recorded.

This type of bioelectrical impedance analyzer was selected because it is much more accurate than the traditional ones (Pietrobelli et al., 2004), showing a good correlation with DXA (Malavolti et al., 2003), and also because it had been previously used successfully in other studies (Colado and Triplett, 2008; Colado et al., 2009b).

### Physical capacity

Three tests of physical capacity were chosen for this study. Subjects performed a protocol warm-up before the evaluation and had a recovery period of 10 minutes between tests. They performed the test at the same time of day before and after the intervention period and in the same order. The knee push-up test (KPU) for total repetitions was used as the measure of upper body muscle endurance (American College of Sports Medicine, 1999). This test was performed from the bent-knee position and was not timed. In contrast, the 60-s squat test (ST) was used as a measure of lower body muscle endurance (American College of Sports Medicine, 1999). The maximal number of repetitions were performed in 1 min with a thigh position parallel to the floor at the bottom of the range of movement and without carrying out assistance movements using other body segments. The abdominal crunch (partial curl-up) test (CT), or Canadian crunch test, was employed for assessing abdominal muscle endurance. It consists of the number of repetitions completed in 3 min at a pace of 25 repetitions per minute with a cadence marked by a metronome of 50 beats per minute. This test must be stopped when the subject cannot continue, when there is poor technique for more than two consecutive repetitions or when the maximum number of repetitions (i.e., 75) is reached. The physical tests took place in the morning, approximately 1.5 h after the subjects had consumed their normal breakfast and after approximately 8 h of sleep. All subjects were verbally encouraged throughout all physical tests. Each test was supervised by the same examiner, with two reference examiners who were present to monitor strict compliance with the protocol.

### Training protocol

*The women* were taught the techniques for each of the different exercises in two sessions before the start of the training program, following established criteria for body position, range of motion and respiration (Colado and García-Massó, 2009). Moreover, the speed of movement was standard for the land-based exercises, using an individualized and slow pace (for example, 2 seconds concentric, 4 seconds eccentric) in the ADIDF exercises because the speed of these exercises affects their intensity, that is, the faster the execution speed, the greater the intensity. During these sessions, the women also became familiar with the method of controlling intensity through the combined use of the number of repetitions performed and perceived exertion through the OMNI-RES-AM (Robertson et al., 2003). This method of changing the intensity of the exercises with EBs or ADIDF has been previously described (Colado and Triplett, 2008; Colado et al., 2008; Colado and Triplett, 2009; Colado et al., 2009a; Colado et al., 2009b). In each of the familiarization sessions, the subjects learned the techniques for six of the 12 exercises in the program. We also carried out a test of 20 maximum repetitions (MR) to determine the starting point of every subject, as indicated by previous authors (Colado and Triplett, 2008; Colado et al., 2009b).

The EBG used Thera-Band^®^ elastic resistance bands of a moderate intensity (green color) with a length of 1 meter. Every subject always trained with the same EB. The WMG used Switching Machines (TECA Srl, Ortona, Italy) and the ADIDFG chose between three different ADIDF for the upper body (Aquagloves ™, Aqua exercisers ™ paddle, and Hydro-Tone Bells ™-Sprint Aquatics, San Luis Obispo, CA USA-) and two for the lower body (Aquafins ™ Hydro-tone or Boots ™-Sprint Aquatics, San Luis Obispo, CA, USA). Switching Machines are a type of weight machines on which the exercisers can train in standing position and without points of corporal support or with a much reduced quantity of points of corporal support. Each device generated different intensities, with the objective of having each subject choose the intensity that would allow them to reach the number of repetitions and the pre-established value on the OMNI scale.

The periodized training program lasted 10 weeks with 2 sessions per week. All the subjects adhered strictly to the program, with a minimum of 95% attendance at training sessions. Six exercises that involved the major muscle groups of the whole body in an agonistic manner were always used, with a total of 20 repetitions performed at an intensity of 5 or “somewhat hard” OMNI-RES AM for the first 4 weeks of adaptation and an intensity of 7 or “hard” OMNIRES AM for the next 6 weeks. In the first 4 weeks, 2 sets were performed for the lower and 1 set for the upper extremities; from weeks 5 to 8, the number of sets was equalized for the upper and lower body, and for weeks 9 and 10, the number of sets was increased to 3. Between exercises, there was an active recovery period of 30 seconds consisting of gentle jogging. As this study was not intended to improve cardiovascular endurance, this was not addressed in the training program. In order to increase motivation, the order of the exercises and how they were performed was changed every week in the same way for both groups. The proposal of exercises and combination between them that was indicated in previous studies by Colado and Triplett (2008) and Colado et al. (2009b) was used for applying this conditioning program. The sessions were always monitored by the same qualified technicians and were also supervised by trained monitors in order to corroborate the methodology, performance, materials, room conditions and program adherence. Warm-up and cool-down protocols were designed and followed by both groups. Due to the design and monitoring of the training protocol, none of the women were injured during the training program.

### Statistical analysis

The homogeneity of the dependent variables was checked using Levene’s test, and their normality was also evaluated by means of Kolgomorov-Smirnov statistics. Descriptive statistics were then calculated and are expressed as mean (SEM). One-way repeated-measures analyses of variance (ANOVA) were used to determine the effect of each intervention in those variables that passed the normality and homoscedasticity criteria. When differences were found, a Bonferroni post-hoc analysis was performed. A non-parametric Wilcoxon test for paired samples was used to find differences within groups in the variables that did not corroborate the assumptions (i.e., ST and KPU).

In addition, a Kruskal-Wallis test was applied to establish differences between groups at the pre- and post-test for these variables. When differences were found, a post-hoc analysis was performed by means of a Mann-Whitney U test for unrelated samples. The level of significance for all analyses was set at 0.05.

## Results

By comparing the values of all variables between the groups in the pre-test, significant differences were only found in the ST (p = 0.037). These differences existed between the WMG and EBG (p = 0.006) and between the WMG and ADIDFG (p = 0.006), with higher values for the WMG group. These differences disappeared in the post-test.

**Figure 1:**
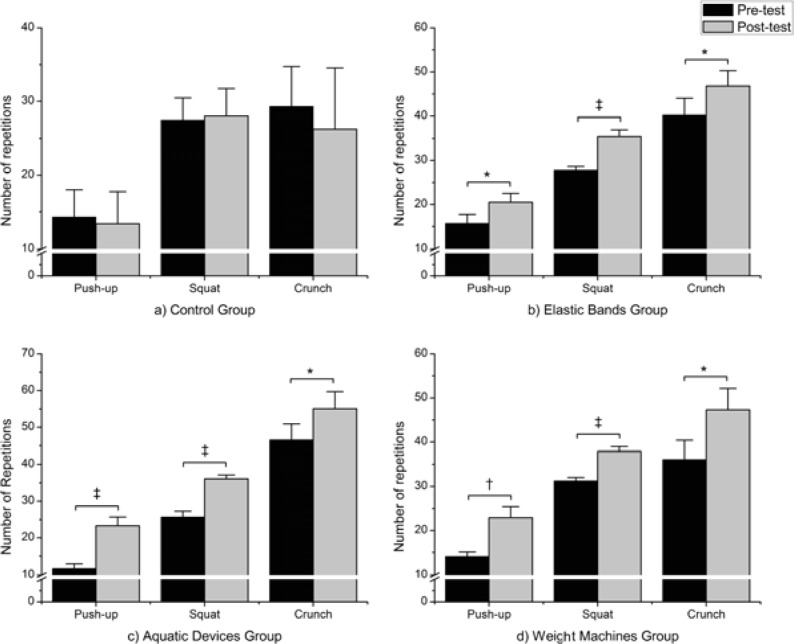
*Comparisons between the groups in the post-test showed differences in FM between the EBG and the WMG (p = 0.04)*,

Comparisons between the groups in the post-test showed differences in FM between the EBG and the WMG (p = 0.04), with lower values for the group that trained with machines (see Figure 1). There were also differences between groups in the variable CT. The CG managed to complete significantly fewer crunches than the EBG (p = 0.05) and ADIDFG (p = 0.004). We also observed significantly higher results for the variable ST in the ADIDFG (p = 0.05) and WMG (p = 0.021) compared to the CG in the post-test.

There were differences in FM, FFM, FFM-LA, FFM-RA, KPU, ST and CT when comparing each group in the pre-and post-test. The ADIDFG showed improvements in FM, FFM-LA, FFM-RA, KKPU, ST and CT. The EBG and WMG also improved with respect to their FM, FFM, FFM-LA, FFM-RA, KPU, ST and CT. Table 2 shows the results of the effects of intervention in morphological variables.

## Discussion

This is the first work that uses three different devices (EBs, WMs and ADIDF) to conduct an equivalent program for strength training in postmenopausal women with the aim of improving their physical fitness and body composition. There are no previous studies comparing the effects of a training program using these three devices. The great difficulty in designing a methodology that allows the control of the intensity of exercises performed with any of these devices has so far hindered the completion of such studies. Until now, several studies had found great difficulty in controlling the intensity of exercises performed with EBs (Patterson et al., 2001; Thomas et al., 2005) and ADIDF (Petrick et al., 2001; Pöyhönen et al., 2001). In the case of EBs, this is due to different coefficients of elongation of the rubbers and their modification during use because this could provoke different levels of resistance (Thomas et al., 2005) as well as it also could provoke a difficulty in evaluating and therefore in comparing them (Patterson et al., 2001). Regarding ADIDF exercises, the main difficulties are found when attempting to generate enough resistance intensity and to maintain this intensity (Petrick et al., 2001; Pöyhönen et al., 2001). These problems have been resolved by using the OMNI-RES-AM combined with the target number of repetitions as a means to control intensity. In addition, the width of the grip of the EBs and the speed of execution, along with the size and position of the ADIDF hydrodynamics in the exercises that employ such devices, were the parameters that were used to accommodate the potential resistance of each subject. This new system has been used in recent studies on the same segment of the population and has been considered effective (Colado and Triplett, 2008; Colado et al., 2009b). However, none of these previous studies had made a comparison of the three devices.

The results obtained in our study suggest that there are minimal differences in the effectiveness of the use of ADIDF, EBs or WMS to improve physical capacity and body composition in postmenopausal women. The different resources for strength training that have been used in this study have shown the potential to cause improvements in the post-test compared to the pre-test. Similar results have already been published, such as the study by Colado et al. (2009b), which compared ADIDF and EBs. The ADIDFG used here found significant increases of 98.04%, 40.26% and 18.18% in the number of repetitions for push-ups, squats and crunches, respectively. In addition, there was a significant reduction of FM (2.57%) and FFM tended to increase (0.51%). Several studies have also found improvements in physical capacity and body composition through training in an aquatic environment (Colado et al., 2009a; Tsourlou et al., 2006; Volaklis et al., 2007). However, only one of these studies used methods of assessing these parameters similar to those used in our work, including selecting a sample from the same sector of the population (Colado et al., 2009b). That study found a higher improvement in various parameters using a program similar to the one described here. However, these differences could be because their training program had a duration and training volume much higher than ours (i.e., 24 weeks).

It should also be noted that the subjects in the ADIDFG showed increases in upper limb FFM (3.7% and 2.06% for the left and right sides, respectively) with no differences in the FFM of the lower limbs. When considering that the other experimental groups also failed to show significant increases in FFM in the legs, one can conclude that the intensity and volume of our program were not sufficient to cause these changes, as has been noted in previous studies (Colado et al., 2009b). In turn, this could have influenced the fact that there were no improvements in the FFM in the ADIDFG, yet there were improvements in the EBG and WMG, because the increase in muscle mass of the upper limbs does not require a sufficient percentage of the total weight for the appearance of a general improvement in the FFM. This happened in the other two groups because although they did not show significant increases in FFM of the lower limbs, there were no improvements that were superior to those of the ADIDFG.

Programs with the other two devices found similar improvements. The EBG showed a significant increase in the post-test of 30.62%, 16.27% and 27.4% in the number of pushups, crunches and squats respectively. In addition, there was a decrease in FM of 1.93% and a 1.15% increase in FFM; both were significant changes. Furthermore, the group training with WMs managed in the post-test to significantly increase the number of push-ups (62.62%), crunches (31.11%) and squats (21.14%) completed and also showed increases in the FFM (2.52%) and decreases in body fat (5.15%). In addition, there were few differences between the effectiveness of the various devices for strength training when comparing the results of the post-test. One of the most striking results was the greater reduction of body fat using WMs in contrast to EBs as previous studies have failed to observe these differences (Colado and Triplett, 2008). It is possible that the non-significant divergences found between these groups in the pre-test (i.e. 28.39 in the EBG group and 22.34 in the WMG group) are responsible for these results. Finally, by observing the percentage reduction in FM in both groups, the EBs reduced its body fat by 1.93% and the WMG by 5.15%. On the other hand, the ADIDFG showed a 2.5% reduction in body fat. Consequently, it may be suggested that programs that use WMs can be more effective in reducing FM in the short term, but further studies are needed to confirm this hypothesis because there were small differences regarding this variable between the groups in the pre-test.

Although it could be considered that our results favor training with WMs to improve body composition, this was not observed with respect to variables related to physical capacity. By observing the improvements of the three groups, the most pronounced increases with respect to the number of flexions and sit-ups that they were able to perform are seen in the ADIDFG (98.2% and 40.26% compared to 62.62% and 21.14% for the WMG and 30.6% and 27.4% for the EBG). These results support previous results characterizing strength training in an aquatic environment to be at least equivalent to land-based training in terms of increasing muscle strength (Colado et al., 2009b).

However, it should be noted that it is possible that this study is committing type II errors in some of the analysis relating to the comparisons between groups, because there were no significant differences between the GC and the experimental groups in the post-test for most of the variables. Thus, it is possible that apart from suggesting differences between the intervention groups and the GC in the post-test, this also increases the number of significant differences in the different variables between experimental groups.

In summary, this study has important implications related to both the possible practical applications of these data as well as the need to further continue this line of research. Concerning the practical applications, we have shown that training using ADIDF is as effective as training using EBs or WMs to improve physical capacity in postmenopausal women, and also results in improvements in the body composition of the subjects. In addition, our study continues to support the use of the OMNI-RES-AM along with the number of target repetitions as an effective tool to control the intensity of the exercises, as important adaptations have been achieved by using this scale. However, it remains necessary for further studies to address this issue specifically. Furthermore, as already suggested in previous studies, this resource can be of great help when devices are used for strength training that cannot be adjusted according to the amount of resistance provided (Colado et al., 2010). Finally, it should also be noted that due to limitations on the sample size when trying to obtain high statistical power for the comparisons between groups, it may be of interest to implement research projects that address these shortcomings and that supplement the results reported in this investigation. However, despite this limitation, it must be highlighted the big effort to equalize the three treatment groups and accordingly we think that this article can contribute positively to the literature in this area.

Training with ADIDF is effective in improving the body composition and physical capacity of postmenopausal women in the short term. As expected, the land-based exercises performed with either of the two devices used in this regimen also achieved good results. Our results support a diversified exercise prescription regarding the scheduling of training exercises that employ the use of resistance so that a methodological approach as shown here can be complemented with effective training sessions and different devices. Thus, the training process will be facilitated with a greater variety of stimuli and a better ability to accommodate both the individual and the resources available.

## Figures and Tables

**Table 1:** Subject characteristics

	CG (n=10)	WMG (n=14)	EBG (n=21)	ADIDFG (n=17)
Age (years)	53.9 (0.59)	51.07 (1.82)	54.14 (0.63)	54.71 (0.45)
Weight (kg)	65.91 (3.08)	62.38 (2.57)	69.23 (2.23)	67.62 (2.21)

Data are expressed as mean (SEM).

CG= control group; WMG= weight machines group; EBG= elastic bands group; ADIDFG= aquatic devices that increase the drag force group.

**Table 2:** Effects of intervention in morphological variables

		Fat mass (Kg)	Fat free mass (Kg)	Fat free mass of right leg (Kg)	Fat free mass of left leg (Kg)	Fat free mass of right arm (Kg)	Fat free mass of left arm (Kg)
Control	Pre-test	26,03 (2,42)	39,88 (0,89)	6,32 (0,15)	6,28 (0,15)	1,84 (0,06)	1,89 (0,07)
	Post-test	25,72 (2,52)	40,06 (0,96)	6,34 (0,15)	6,28 (0,15)	1,9 (0,07)	1,9 (0,07)
Elastic bands	Pre-test	28,39 (1,62)	40,84 (0,69)	6,52 (0,12)	6,46 (0,12)	1,89 (0,04)	1,96 (0,05)
	Post-test	27,84 (1,54)^*a^	41,31 (0,7)^*^	6,55 (0,12)^*^	6,49 (0,12)	1,96 (0,03)^†^	1,99 (0,05)^*^
Aquatic devices	Pre-test	26,08 (1,7)	41,55 (0,61)	6,48 (0,1)	6,42 (0,09)	1,89 (0,03)	1,94 (0,05)
	Post-test	25,41 (1,67)^*^	41,76 (0,64)	6,43 (0,11)	6,42 (0,11)	1,96 (0,03)^†^	1,98 (0,05)^*^
Weight machines	Pre-test	22,34 (1,62)	40,06 (1,06)	6,35 (0,19)	6,27 (0,18)	1,85 (0,05)	1,85 (0,06)
	Post-test	21,19 (1,53)^†a^	41,07 (1,01)^‡^	6,43 (0,17)	6,33 (0,16)	1,93 (0,06)^‡^	1,92 (0,06)^‡^

* Significant difference between pre and post test (p≤0.05)

† Significant difference between pre and post test (p≤0.005)

‡ Significant difference between pre and post test (p≤0.001)

a Significant difference between groups in the post test (p≤0.05)

## References

[b1-jhk-32-185] American College of Sports Medicine (1999). Handbook for the evaluation and prescription of exercise.

[b2-jhk-32-185] American College of Sports Medicine (2009). Progression models in resistance training for healthy adults. Medicine and Science in Sports and Exercise.

[b3-jhk-32-185] Bemben DA, Bemben MG (2000). Effects of resistance exercise and body mass index on lipoproteinlipid patterns of postmenopausal women. Journal of Strength and Conditioning Research.

[b4-jhk-32-185] Bemben DA, Fetters NL, Bemben MG, Nabavi N, Koh ET (2000). Musculoskeletal responses to high- and low-intensity resistance training in early postmenopausal women. Medicine and Science in Sports and Exercise.

[b5-jhk-32-185] Bushman BA, Flynn MG, Andres FF, Lambert CP, Taylor MS, Braun WA (1997). Effect of 4 wk of deep water run training on running performance. Medicine and Science in Sports and Exercise.

[b6-jhk-32-185] Chulvi-Medrano I, Colado JC, Pablos C, Naclerio F, García-Massó X (2009). A lower-limb training program to improve balance in healthy elderly women using the T-Bow^®^ device. The Physician and Sportsmedicine.

[b7-jhk-32-185] Colado JC, García-Massó X (2009). Technique and safety aspects of resistance exercises: A systematic review of the literature. The Physician and Sportsmedicine.

[b8-jhk-32-185] Colado JC, Garcia-Masso X, Pellicer M, Alakhdar Y, Benavent J, Cabeza-Ruiz R (2010). A comparison of elastic tubing and isotonic resistance exercises. International Journal of Sports Medicine.

[b9-jhk-32-185] Colado JC, Tella V, Triplett NT (2008). A method for monitoring intensity during aquatic resistance exercises. Journal of Strength and Conditioning Research.

[b10-jhk-32-185] Colado JC, Tella V, Triplett NT, Gonzalez LM (2009a). Effects of a short-term aquatic resistance program on strength and body composition in fit young men. Journal of Strength and Conditioning Research.

[b11-jhk-32-185] Colado JC, Triplett NT (2008). Effects of a short-term resistance program using elastic bands versus weight machines for sedentary middle-aged women. Journal of Strength and Conditioning Research.

[b12-jhk-32-185] Colado JC, Triplett NT (2009). Monitoring the intensity of aquatic resistance exercises with devices that increase the drag force: an update. Strength and Conditioning Journal.

[b13-jhk-32-185] Colado JC, Triplett NT, Tella V, Saucedo P, Abellan J (2009b). Effects of aquatic resistance training on health and fitness in postmenopausal women. European Journal of Applied Physiology.

[b14-jhk-32-185] Colado JC, Garcia-Masso X, Triplett NT, Flandez J, Borreani S, Tella V (2011). Concurrent Validation of the OMNI-Resistance Exercise Scale of Perceived Exertion with Thera-Band® Resistance Bands. Journal of Strength and Conditioning Research.

[b15-jhk-32-185] Dishman RK, Washburn RA, Heath GW (2004). Physical Activity Epidemiology.

[b16-jhk-32-185] Dixon CB, Deitrick RW, Pierce JR, Cutrufello PT, Drapeau LL (2005). Evaluation of the BOD POD and leg-to-leg bioelectrical impedance analysis for estimating percent body fat in national collegiate athletic association division III collegiate wrestlers. Journal of Strength and Conditioning Research / National Strength and Conditioning Association.

[b17-jhk-32-185] Elliott KJ, Sale C, Cable NT (2002). Effects of resistance training and detraining on muscle strength and blood lipid profiles in postmenopausal women. British Journal of Sports Medicine.

[b18-jhk-32-185] Fahlman MM, Boardley D, Lambert CP, Flynn MG (2002). Effects of endurance training and resistance training on plasma lipoprotein profiles in elderly women. The Journals of Gerontology.

[b19-jhk-32-185] Frangolias DD, Rhodes EC, Taunton JE (1996). The effects of familiarity with deep water running on maximal oxygen consumption. Journal of Strength and Conditioning Research.

[b20-jhk-32-185] García-Massó X, Colado JC (2010). Muscular activity of the posterior deltoid during swimming vs. resistance exercises on water and dry-land: A case study. Internation Journal of Aquatic Research and Education.

[b21-jhk-32-185] Katula JA, Sipe M, Rejeski WJ, Focht BC (2006). Strength training in older adults: An empowering intervention. Medicine and Science in Sports and Exercise.

[b22-jhk-32-185] Malavolti M, Mussi C, Poli M, Fantuzzi AL, Salvioli G, Battistini N, Bedogni G (2003). Cross-calibration of eight-polar bioelectrical impedance analysis versus dual-energy X-ray absorptiometry for the assessment of total and appendicular body composition in healthy subjects aged 21–82 years. Annals of Human Biology.

[b23-jhk-32-185] Martel GF, Harmer ML, Logan JM, Parker CB (2005). Aquatic plyometric training increases vertical jump in female volleyball players. Medicine and Science in Sports and Exercise.

[b24-jhk-32-185] Miller MG, Berry DC, Bullard S, Giklers R (2002). Comparisons of land-based and aquatic-based plyometric programs during an 8-week training period. J Sport Rehabil.

[b25-jhk-32-185] Patterson RM, Stegink Jansen CW, Hogan HA, Nassif MD (2001). Material properties of thera-band tubing. Physical Therapy.

[b26-jhk-32-185] Petrick M, Paulsen T, George J (2001). Comparison between quadriceps muscle strengthening on land and in water. Physiotherapy.

[b27-jhk-32-185] Pietrobelli A, Rubiano F, St-Onge MP, Heymsfield SB (2004). New bioimpedance analysis system: Improved phenotyping with whole-body analysis. European Journal of Clinical Nutrition.

[b28-jhk-32-185] Ploutz-Snyder LL, Giamis EL (2001). Orientation and familiarization to 1RM strength testing in old and young women. J Strength Cond Res.

[b29-jhk-32-185] Pöyhönen T, Keskinen KL, Kyrolainen H, Hautala A, Savolainen J, Malkia E (2001). Neuromuscular function during therapeutic knee exercise under water and on dry land. Archives of Physical Medicine and Rehabilitation.

[b30-jhk-32-185] Robertson RJ, Goss FL, Rutkowski J, Lenz B, Dixon C, Timmer J, Andreacci J (2003). Concurrent validation of the OMNI perceived exertion scale for resistance exercise. Medicine and Science in Sports and Exercise.

[b31-jhk-32-185] Robinson LE, Devor ST, Merrick MA, Buckworth J (2004). The effects of land vs. aquatic plyometrics on power, torque, velocity, and muscle soreness in women. Journal of Strength and Conditioning Research / National Strength and Conditioning Association.

[b32-jhk-32-185] Takeshima N, Rogers ME, Watanabe E, Brechue WF, Okada A, Yamada T, Hayano J (2002). Water-based exercise improves health-related aspects of fitness in older women. Medicine and Science in Sports and Exercise.

[b33-jhk-32-185] Thomas M, Muller T, Busse MW (2005). Quantification of tension in thera-band and cando tubing at different strains and starting lengths. The Journal of Sports Medicine and Physical Fitness.

[b34-jhk-32-185] Triplett NT, Colado JC, Benavent J, Alakhdar Y, Madera J, Gonzalez LM, Tella V (2009). Concentric and impact forces of single-leg jumps in an aquatic environment versus on land. Medicine and Science in Sports and Exercise.

[b35-jhk-32-185] Tsourlou T, Benik A, Dipla K, Zafeiridis A, Kellis S (2006). The effects of a twenty-four-week aquatic training program on muscular strength performance in healthy elderly women. Journal of Strength and Conditioning Research.

[b36-jhk-32-185] Volaklis KA, Spassis AT, Tokmakidis SP (2007). Land versus water exercise in patients with coronary artery disease: Effects on body composition, blood lipids, and physical fitness. American Heart Journal.

